# Supplementary material for: Spatial and temporal variation in respiratory syncytial virus (RSV) subtype RNA in wastewater and relation to clinical specimens
Source: mSphere. 2024 Jun 27;9(7):e00224-24. doi: 10.1128/msphere.00224-24 (PMC11288019; doi:10.1128/msphere.00224-24)
Supplement: Supplemental Material — Supplemental methods, results, figures, and tables. [file msphere.00224-24-s0001.pdf]

Supporting Material

Spatial and temporal variation in respiratory syncytial virus (RSV) subtype RNA in wastewater, and relation to clinical specimens

Winnie Zambrana[1], ChunHong Huang[2], Daniel Solis[2], Malaya K. Sahoo[2], Benjamin A. Pinsky[2,3], Alexandria B. Boehm[1*]

1. Department of Civil & Environmental Engineering, Stanford University, 473 Via Ortega, Stanford, CA, USA, 94305
2. Department of Pathology, Stanford University School of Medicine, Stanford CA, 94305
3. Department of Medicine, Division of Infectious Diseases and Geographic Medicine, Stanford University School of Medicine, Stanford CA, 94305

*Corresponding author
Alexandria B. Boehm, aboehm@stanford.edu, 650-724-9128

1. Materials and Methods

Study Design

The samples in this study are part of a larger COVID-19 wastewater surveillance effort which, alongside several viruses, measures total respiratory syncytial virus (RSV) (sum of RSV A and RSV B, not subtyped) routinely[1]. Total RSV was measured in fresh samples alongside PMMoV and BCoV, as described in the main text. Sequences of primers and probes for Total RSV used in the larger epidemiology effort can be found in **Table S1**. Total RSV results are not reported in this study, and they were only used for original sample selection and storage impact assessment.

Procedures

The primers and probes sequences used in the study and their corresponding references can be found in **Table S1-S2**.

2. Results

Concentrations of RSV A and RSV B at each wastewater treatment plant are shown in **Figure S1**, and the proportion of RSV A to Total RSV at each wastewater treatment plant is shown in **Figure S2**.

QA/QC.

The Environmental Microbiology Minimal Information (EMMI) guidelines checklist can be found in **Figure S3**, and examples of fluorescent plots for this study can be found in **Figure S4**. To alleviate inhibition, dewatered solid samples were resuspended at a concentration of 0.75 mg (wet weight)/ml which has been shown to minimize inhibition of measurements[2]. Additionally, sample extracts were treated with Zymo One-Step PCR inhibitor removal columns (Zymo Research, Irvine, CA).

3. References

(1) Zulli, A.; Varkila, M. R. J.; Parsonnet, J.; Wolfe, M. K.; Boehm, A. B. Observations of Respiratory Syncytial Virus (RSV) Nucleic Acids in Wastewater Solids Across the United States in the 2022–2023 Season: Relationships with RSV Infection Positivity and Hospitalization Rates. *ACS EST Water* **2024**. https://doi.org/10.1021/acsestwater.3c00725.

(2) Huisman, J. S.; Scire, J.; Caduff, L.; Fernandez, -Cassi Xavier; Ganesanandamoorthy, P.; Kull, A.; Scheidegger, A.; Stachler, E.; Boehm, A. B.; Hughes, B.; Knudson, A.; Topol, A.; Wigginton, K. R.; Wolfe, M. K.; Kohn, T.; Ort, C.; Stadler, T.; Julian, T. R. Wastewater-Based Estimation of the Effective Reproductive Number of SARS-CoV-2. *Environ. Health Perspect.* **2022**, *130* (5), 057011. https://doi.org/10.1289/EHP10050.

(3) Borchardt, M. A.; Boehm, A. B.; Salit, M.; Spencer, S. K.; Wigginton, K. R.; Noble, R. T. The Environmental Microbiology Minimum Information (EMMI) Guidelines: qPCR and dPCR Quality and Reporting for Environmental Microbiology. *Environ. Sci. Technol.* **2021**, *55* (15), 10210–10223. https://doi.org/10.1021/acs.est.1c01767.

(4) Borchardt, M.; Boehm, A.; Wigginton, K.; Noble, R.; Salit, M.; Spencer, S. Environmental Microbiology Minimal Information Checklist. **2023**. https://doi.org/10.25740/TM549RG8921.

(5) Boehm, A. B.; Hughes, B.; Duong, D.; Chan-Herur, V.; Buchman, A.; Wolfe, M. K.; White, B. J. Wastewater Concentrations of Human Influenza, Metapneumovirus, Parainfluenza, Respiratory Syncytial Virus, Rhinovirus, and Seasonal Coronavirus Nucleic-Acids during the COVID-19 Pandemic: A Surveillance Study. *Lancet Microbe* **2023**, *4* (5), e340–e348. https://doi.org/10.1016/S2666-5247(22)00386-X.

(6) Haramoto, E.; Kitajima, M.; Kishida, N.; Konno, Y.; Katayama, H.; Asami, M.; Akiba, M. Occurrence of Pepper Mild Mottle Virus in Drinking Water Sources in Japan. *Appl. Environ. Microbiol.* **2013**, *79* (23), 7413–7418. https://doi.org/10.1128/AEM.02354-13.

(7) Zhang, T.; Breitbart, M.; Lee, W. H.; Run, J.-Q.; Wei, C. L.; Soh, S. W. L.; Hibberd, M. L.; Liu, E. T.; Rohwer, F.; Ruan, Y. RNA Viral Community in Human Feces: Prevalence of Plant Pathogenic Viruses. *PLOS Biol.* **2005**, *4* (1), e3. https://doi.org/10.1371/journal.pbio.0040003.

(8) Decaro, N.; Elia, G.; Campolo, M.; Desario, C.; Mari, V.; Radogna, A.; Colaianni, M. L.; Cirone, F.; Tempesta, M.; Buonavoglia, C. Detection of Bovine Coronavirus Using a TaqMan-Based Real-Time RT-PCR Assay. *J. Virol. Methods* **2008**, *151* (2), 167–171. https://doi.org/10.1016/j.jviromet.2008.05.016.
(9) Hughes, B.; Duong, D.; White, B. J.; Wigginton, K. R.; Chan, E. M. G.; Wolfe, M. K.; Boehm, A. B. Respiratory Syncytial Virus (RSV) RNA in Wastewater Settled Solids Reflects RSV Clinical Positivity Rates. *Environ. Sci. Technol. Lett.* **2022**, *9* (2), 173–178. https://doi.org/10.1021/acs.estlett.1c00963.

Figures

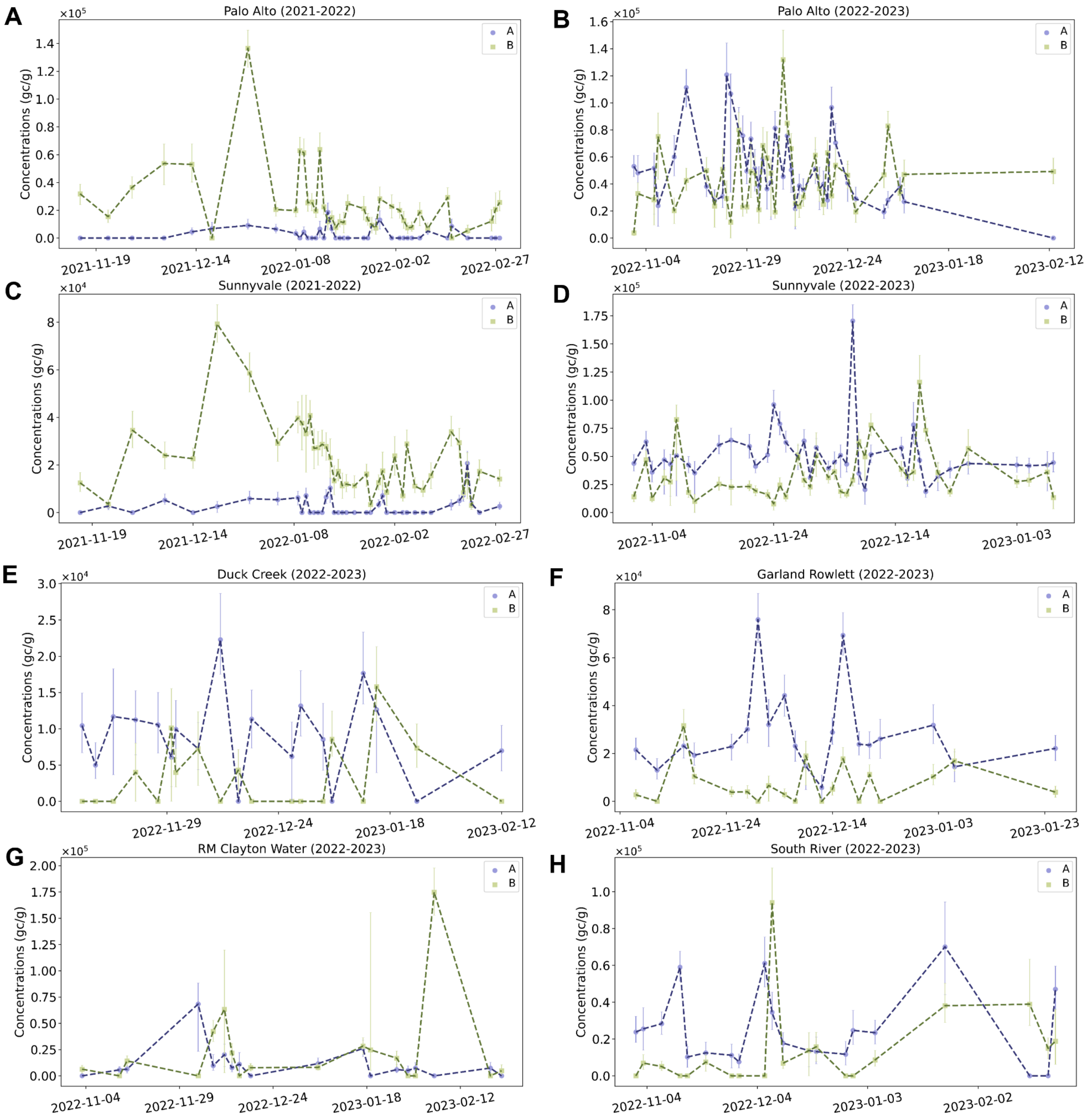

Figure S1. RSV A (purple) and RSV B (green) concentrations for all WWTPs included in the study. Palo Alto Season 1 [San Jose, CA] (A) ; Palo Alto Season 2 [San Jose, CA] (B); Sunnyvale Season 1 [San Jose, CA] (C); Sunnyvale Season 2 [San Jose, CA] (D); Duck Creek Season 2 [Dallas, TX] (E); Garland Rowlett Season 2 [Dallas, TX] (F); RM Clayton Water Season 2 [Atlanta, GA] (G); South River Season 2 [Atlanta, GA] (H). Error bars represent the standard deviation as the total error reported by the ddPCR instrument.

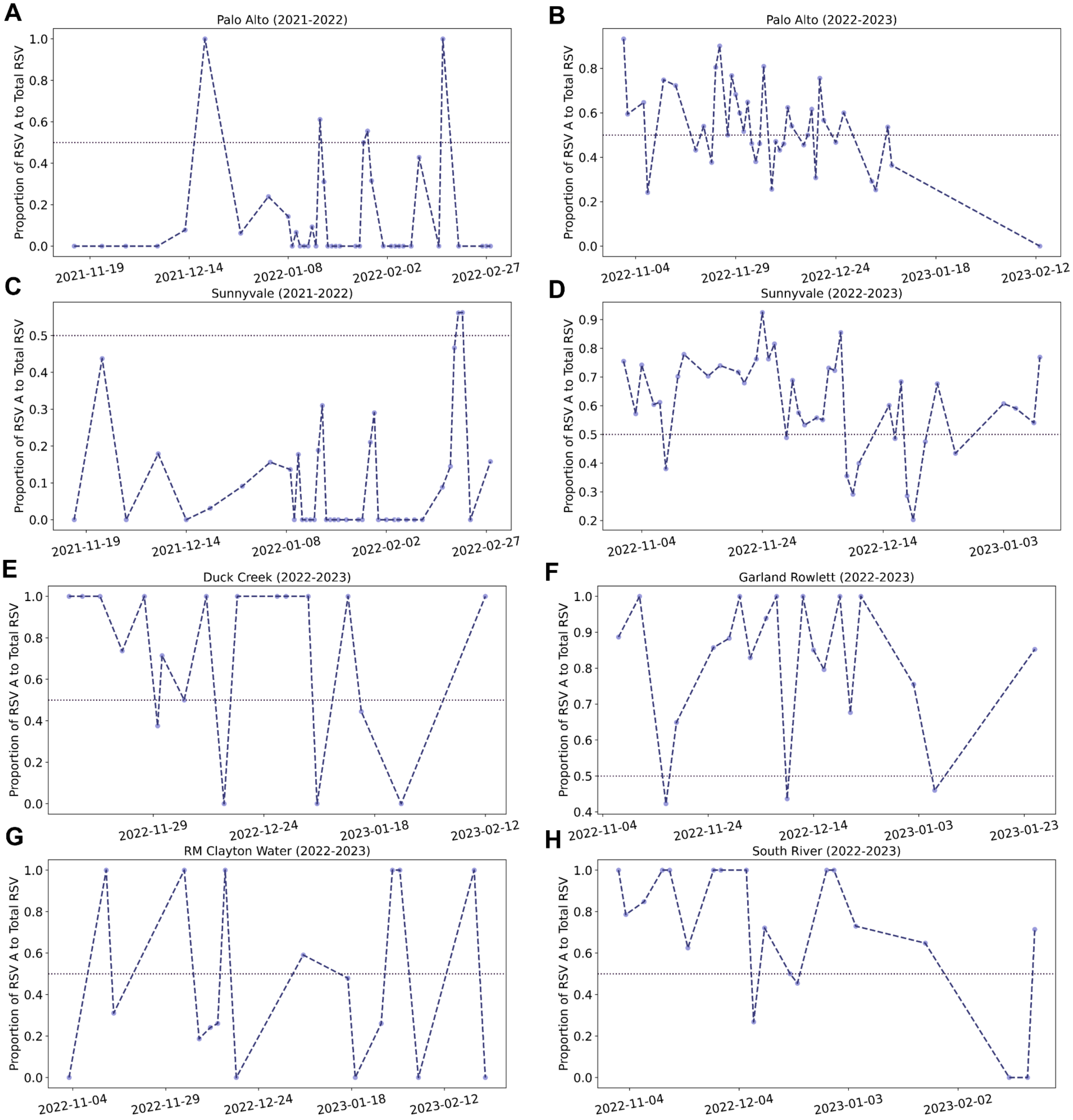

Figure S2. Proportion of RSV A to total RSV for all WWTPs included in the study. Palo Alto Season 1 [San Jose, CA] (A) ; Palo Alto Season 2 [San Jose, CA] (B); Sunnyvale Season 1 [San Jose, CA] (C); Sunnyvale Season 2 [San Jose, CA] (D); Duck Creek Season 2 [Dallas, TX] (E); Garland Rowlett Season 2 [Dallas, TX] (F); RM Clayton Water Season 2 [Atlanta, GA] (G); South River Season 2 [Atlanta, GA] (H). Dashed horizontal line indicates a proportion of 0.5.

Study Description	Environmental Sampling	Sample Treatment	Sample Reduction	Nucleic-acid Extraction	Reverse Transcription	PCR Amplification	Analysis
Study name: RSV A/B in w<br>Date: January 10-2024<br>Completed by: Winnie Zam	Notes: As described in text	Notes: Performed for wastewater solids samples - Samples were frozen and stored as described in the text. Information on the effect on samples through total RSV and PMMoV concentrations provided.	Notes: Performed for wastewater solids samples - Centrifugation and resuspension as described n text	Notes: As described in text	Notes: Performed -As described in text	Notes: As described in text. No specific control just for the PCR step. Sample extracts were further purified using the Zymo One-Step PCR inhibitor removal columns (Zymo Research, Irvine, CA).	Notes: As described in text.

Control Checklist	Environmental Sampling	Sample Treatment	Sample Reduction	Nucleic-acid Extraction	Reverse Transcription	PCR Amplification	
Step performed	☑	☑	☑	☑	☑	☑	
Step has control info	☐	☐	☐	☑	☑	☐	Negative controls
# of control replicates	NA	NA	NA	2	2	NA	
Control result reported	☐	☐	☐	☑	☑	☐	
Method for handling failed controls described	☐	☐	☐	☑	☑	☐	
Step has control info	☐	☐	☐	☐	☐	☐	Positive controls
Control identity described	☐	☐	☐	☑	☑	☐	
Control quantification method described	☐	☑	☐	☑	☑	☐	
# control replicates	NA	NA	NA	10	2	NA	
Control result reported	☐	☑	☐	☑	☑	☐	
Method for handling failed controls described	☐	☐	☐	☑	☑	☐	

Process checklist

Environmental Sampling	
Sample procedure	☑
Number of samples	☑
Sample amount, mean, range	☑
Sampling locations, dates, times	☑
Sample storage conditions	☑
**Sample Treatment**	
Treatment procedure	☑
Reagents	☐
**Sample Reduction**	
Reduction procedure	☑
Reagents	☐
Concentration factor	☐

Nucleic-acid Extraction	
Extraction procedure	☑
Volume or mass extracted, volume or mass obtained	☐
Extract storage conditions	☑
**Reverse Transcription**	
One- or two-step	☑
cDNA storage conditions (if 2 step)	☐
Reaction temperatures and times	☑
Reaction reagents and concentrations	☑
Priming method	☑
Reaction volume, added template amount	☑
RT efficiency assessment procedure (if 2-step)	☐
RT control description (if two-step)	☐
RT efficiency reported (if 2-step)	☐

qPCR or dPCR	
Target gene name, amplicon length	☑
Thermocycling temp and times	☑
Master mix composition: vendors, concentrations	☑
Additives: vendors, composition	☑
Template amount added, pre-treatment (if any)	☑
Primers: sequences, concentrations, vendors, references	☑
Amplicon confirmation method (probe, melt curve details, etc)	☑
Probe sequence, concentration, vendor, reference	☑
Instrumentation	☑
Inhibition assessment procedure	☑
Inhibition control description (if used)	☐
Number of samples tested and found inhibited	☐
Equivalent volume of sample analyzed	☐

Analysis- dPCR	
Threshold settings	☑
Technical replicates, number, well merging	☑
Partitions measured, number, mean, variance	☑
Partition volume	☑
Target copies per partition, mean, variance	☑
Program used for dPCR analysis	☑
Explanation of control results, example plots	☑
**Analysis- qPCR**	
Technical replicates, number, calculations	☑
Calibration standards, description, source	☑
Method of quantifying standards	☑
Calibration curve slope	☐
Calibration curve R2	☐
Lowest standard measured or 95% LOD	☐
Cq value determination methods	☑

Note to users: This checklist is provided as guidance for best practices for reporting, but is not meant to be prescriptive. Not all items in the check list will apply to all studies. Please see Borchardt et al. The Environmental Microbiology Minimum Information (EMMI) Guidelines: qPCR and dPCR Quality and Reporting for Environmental Microbiology. Environmental Science & Technology, 2021, 55, 15, 10210–10223.

Version 3.0

This version maintained by Borchardt, Boehm, Salit, Noble, Wiggington, Spencer

Date: 18 October 2023

Figure S3. Environmental Microbiology Minimal Information (EMMI) guidelines checklist[3,4].

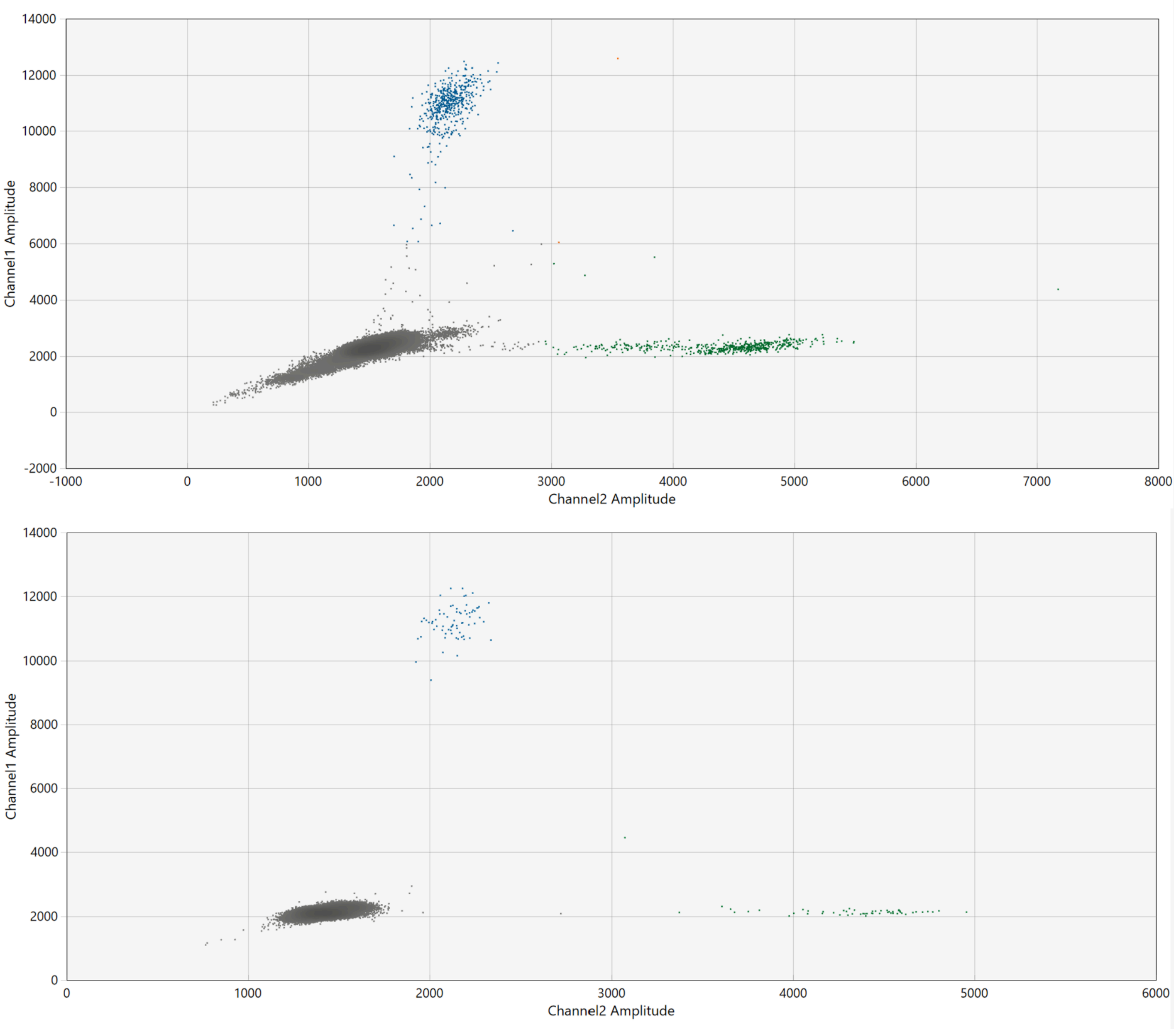

Figure S4. Examples of ddPCR experimental results for the RSV A/B assay. Several wastewater representative samples (top) and positive controls only (bottom).

Tables

Table S1. Primer and probe sequences used in this study for wastewater samples

Target	Forward Primer Sequence	Reverse Primer Sequence	Probe Sequence	Reference
RSV A	AGAGGTGGCAGTAGAGTTGA	CTCCACAACTTGTTCCATTTCTG	ATGGTGCAGGGCAAGTGATG (5' HEX/ZEN/3' IBFQ)	Boehm et al[5]
RSV B	TGACACTCCCAATTATGATGTGC	CCTGTGAATTTATGATTTGCATCTTCAG	ACACCTAAACAAACTATGTGGTATGC (5' FAM/ZEN/3' IBFQ)	Boehm et al[5]
PMMoV[a,b]	GAGTGGTTTGACCTTAACGTTTGA	TTGTCGGTTGCAATGCAAGT	CCTACCGAAGCAAATG (5' HEX/ZEN/3' IBFQ)	Haramoto et al[6] Zhang et al[7]
BCoV[a]	CTGGAAGTTGGTGGAGTT	ATTATCGGCCTAACATACATC	CCTTCATATCTATACACATCAAGTTGTT (5' FAM/ZEN/3' IBFQ)	Decaro et al[8]
Total RSV[a,c]	TCCAGAATAYAGGC-ATGAYTCTCC	CYCTYCTAATYACWGCTGTAAGAC	AACCAAATTAGCAGCAGGA-GATAGATCAG (5′HEX/ZEN/3′IBFQ)	Hughes et al[9]

Note: a = Measured as part of the larger epidemiology effort; b = Additional PMMoV measurements were conducted in singleplex for a subset of samples concurrently with RSV A/B measurements to assess the impact of freeze-thaw cycles; c = Only used in this study to assess the impact of freeze-thaw cycles. The sum of RSV A and RSV B measurements were compared against total RSV measurements with no storage or freeze-thaw cycles.

Table S2. Primer and probes sequences used in this study for clinical samples

Name	Sequence (5'to 3')	Final Concentration
RSV-F Primer	ATGGCTCTTAGCAAAGTCAAGT	400 nM
RSV-R Primer	TGCACATCATAATTRGGAGTRTCA	400 nM
RSV A-Probe	FAM-ACACTCAAC /ZEN/AAAGAT CAACTTCTRTCATCCAGCA-3IABkFQ	200 nM
RSV B-Probe	Cy5-ACATTAAAT /TAO/AAGGATCAG CTGCTGTCATCCAGCA-3IAbRQSp	200 nM
RNase P Forward Primer	AGATTTGGACCTGCGAGCG	100 nM
RNase P Reverse Primer	GAGCGGCTGTCTCCACAAGT	100 nM
RNase P Probe	Cal Fluor 560-TTCTGACCTGAAGGCTCTGCGCG-BHQ-1	50 nM

Note: BHQ, Black Hole Quencher; Cy5, Cyanine-5; FAM, 6-carboxyfluorescein; RSV, Respiratory Syncytial Virus. RSV primers and probes were purchased from Integrated DNA Technologies (IDT; San Diego, California). The probes contain internal quenchers ZEN and TAO in addition to the 3' Iowa Black quenchers FQ (3IABkFQ) and RQ (3IAbRQSp) (all proprietary to IDT). RnaseP primers and probe were purchased from Biosearch Technologies (Petaluma, California).

Table S3. Overall $P_{A,WW}$ results and statistical analysis for the Kruskal-Wallis test

**Group**	**Number of samples (n)**	**Median $P_{A,WW}$**	**$P_{A,WW}$ IQR**	**Kruskal-Wallis P-Value**
**Spatial (n = 157)**				
Santa Clara County, CA[a]	80	0.58	0.26	1.19×10^{-4}*
Dallas, TX	39	0.88	0.34	
Atlanta, GA	38	0.68	0.74	
**Temporal (n = 160)**				
Sunnyvale WWTP (Season 1)	40	0.00	0.16	1.47×10^{-19}*
Palo Alto WWTP (Season 1)	40	0.00	0.11	
Sunnyvale WWTP (Season 2)	40	0.61	0.21	
Palo Alto WWTP (Season 2)	40	0.53	0.21	
**Clinical (n = 160)**				
Wastewater solid samples[a]	80	0.58	0.26	3.49×10^{-19}*
Clinical samples bootstrap[b]	80	0.79	0.03	

Note: $P_{A,WW}$ = proportion of RSV A to total RSV in wastewater solids; IQR =interquartile range ;* = Statistically significant per the Kruskal-Wallis test with a significance level of p =0.006 accounting for the Bonferroni correction; a = The samples included in the groups 'Santa Clara County, CA' and 'Wastewater samples' are the same samples for Sunnyvale WWTP Season 2 and Palo Alto WWTP Season 2 used in the Temporal evaluation; b = The numbers reported on this table were obtained from bootstrapping method (median bootstrap $F_{A, Cl}$ and $F_{A, Cl}$ IQR). The total number of clinical samples tested was 593 with 79% of samples testing positive for RSV A.

Table S4. Statistical analysis results for the Conover-Iman test

Spatial (n = 157)	**Atlanta, GA**	**Dallas, TX**	
**Dallas, TX**	(-2.57)<br>5.49×10^{-3}*	-	
**Santa Clara County, CA**[a]	(1.47)<br>7.13×10^{-2}	(4.49)<br>6.89×10^{-6}*	

Temporal (n = 160)	**Palo Alto (Season 1)**	**Palo Alto (Season 2)**	**Sunnyvale (Season 1)**
**Palo Alto (Season 2)**	(-8.95)<br>5.06×10^{-16}*	-	-
**Sunnyvale (Season 1)**	(0.35)<br>3.62×10^{-1}	(9.30)<br>6.01×10^{-17}*	-
**Sunnyvale (Season 2)**	(-10.89)<br>3.36×10^{-21}*	(-1.94)<br>2.68×10^{-2}	(-11.24)<br>3.68×10^{-22}*

Clinical (n = 160)	**Clinical Samples**[b]
**Wastewater Samples**[a]	(12.67)<br>3.88×10^{-26}*

Note: P-values for the Conover-Iman test with corresponding test statistics in parentheses. The sign of the test statistic is used to indicate the directionality of the differences where a positive test statistic value indicates Column Category> Row Category, negative test statistic value indicates Column Category < Row Category; * = Statistically significant per the Conover-Iman test with a significance level of p =0.006 accounting for the Bonferroni correction; a = The samples included in the groups 'Santa Clara County, CA' and 'Wastewater samples' are the same samples for Sunnyvale WWTP Season 2 and Palo Alto WWTP Season 2 used in the Temporal evaluation; b = The numbers reported on this table were obtained from bootstrapping method (using median bootstrap $F_{A, Cl}$). The total number of clinical samples tested was 593 with 79% of samples testing positive for RSV A.
